# Association of insulin-like growth factor-1 polymorphisms with high myopia in the Chinese population

**Published:** 2012-03-13

**Authors:** Wenjuan Zhuang, Peizeng Yang, Zili Li, Xunlun Sheng, Jingjing Zhao, Shanshan Li, Xueqiu Yang, Wei Xiang, Weining Rong, Yani Liu, Fangxia Zhang

**Affiliations:** 1 The First Affiliated Hospital of Chongqing Medical University, Chongqing Key Laboratory of Ophthalmology, Chongqing, China; 2 Department of Ophthalmology, Affiliated Hospital of Ningxia Medical University, Yinchuan, China; 3 Department of Ophthalmology, People’s Hospital of Ningxia Hui Autonomous Region, Yinchuan, China; 4 Ningxia Medical University, Yinchuan, China

## Abstract

**Purpose:**

The purpose of this study was to determine whether genetic variants in the insulin-like growth factor-1 (*IGF-1*) gene were associated with high myopia in the Chinese population.

**Methods:**

A case-control association study of 421 unrelated Chinese patients with high myopia and 401 control subjects matched in ethnicity and gender was undertaken. Genomic DNA was prepared from peripheral blood. All individuals were genotyped for 7 tag single nucleotide polymorphisms (tSNPs) across the *IGF-1* gene region. Genotypic distribution was tested for Hardy–Weinberg equilibrium. The genotype and allele frequencies were evaluated using the χ^2^ tests. Bonferroni corrections for multiple comparisons were performed.

**Results:**

The polymorphism of rs12423791 showed positive association with extreme myopia (p_allel_=0.006 and p_allel1 recessive model_=0.004, respectively) after Bonferroni correction for multiple testing and the haplotype GC of rs5742629-rs12423791 was also associated with extreme myopia (p=0.033) after 50,000 permutations for multiple comparisons.

**Conclusions:**

The polymorphism of rs12423791 in *IGF-1* may be associated with extreme myopia in the Chinese population and should be investigated further.

## Introduction

High myopia is an acute form of myopia, and is usually defined by the presence of an axial eye length greater than 26 mm or a refractive error of less than −6.00 diopters (D). It is more prevalent in Chinese than Caucasian populations [1,2]. Around 400 million Chinese (33% of the population, 1.5 times the world average) suffer from myopia, of whom about 20% are in the high-degree category. Its prevalence has increased markedly in recent years [3]. High myopia can result in severe ocular morbidity, and extreme myopia (less than −10.00D) exhibits complications that potentially manifest in blindness, such as retinal detachment, macular degeneration, and glaucoma [4]. In Western populations, approximately 25% of decreased vision is caused by myopia, while in some Asian regions, such as China, Singapore and Japan, it accounts for 60%–80% of with decreased vision in young adults [5]. High myopia is now considered the fourth most common cause of irreversible blindness [4]. However, effective treatment methodology and preventive strategies for high myopia have not been fully established. Therefore, it is important to identify the etiology of high myopia.

Myopia is regarded as a complex eye disease affected by both genetic and environmental factors as well as gene-environment interactions [6,7]. While the exact mechanism underlying this abnormal ocular development is still unclear, there is genomic and clinical evidence in different ethnic populations that genetics plays an important role in its development [8-12], especially in high myopia [13]. Insulin-like growth factor-1 (IGF-1) is a polypeptide that plays an important role in cell proliferation, differentiation, and apoptosis. Genetic evidence supporting a role for *IGF-1* in myopia has come from analysis of a large Caucasian cohort where the single nucleotide polymorphisms (SNPs) were significantly associated with high myopia [14]. However, it is not clear whether there is also an association of these SNPs with high myopia in the Chinese population.

## Methods

### Subjects

In this study, 822 unrelated Chinese individuals have been recruited, including 421 cases with high myopia and 401 control subjects. The study was approved by local hospital ethics committees acting in accordance with the Declaration of Helsinki principles. All of the subjects for this study were from the northern regions of China, and informed consent was obtained from each one before the study. Peripheral blood collection and the comprehensive ophthalmic examination were performed, including visual acuity, cycloplegic retinoscopy, and/or autorefraction (Topcon RM-8800; Topcon Corp., Tokyo, Japan), slit-lamp evaluation of the anterior segment (Topcon SL-1E Slit Lamp; Topcon Corp.), ocular movements, intraocular pressure (Topcon NCT CT-80: Topcon Corp.) axial length by contact ultrasound A-scan biometry, and fundus photography (Canon CR6–45NM Fundus Camera; Canon Inc., Tokyo, Japan).

In the high myopia cases, the patients ought to have an axial length greater than 26 mm and a spherical equivalent (SE) refractive error less than −8.0D, or less than −10.0D for extreme myopia, in both eyes. The SEM is defined as sphere plus half-negative cylinder (sphere +[cylinder/2]). Patients with systemic conditions, syndromic disorders such as Strickler or Marfan’s syndromes, or ophthalmic conditions that could predispose to high myopia were excluded. As for the control, 401 healthy unrelated individuals who were ≥40 years of age were recruited. Controls met the following criteria as previously described in other research [15]: (1) had no known ocular disease or other genetic diseases or systemic connective tissue disorders associated with myopia, (2) were without family history of high myopia, (3) had spherical refractive error ranging from −0.50 to +2.00D, and (4) had axial lengths less than 24 mm in both eyes.

### DNA extraction

Genomic DNA was extracted from peripheral blood cells using QIAamp DNA Blood Mini Kit (Qiagen GmbH, Hilden, Germany) according to the manufacturer’s protocol. The isolated DNA was eluted in TE buffer (10mM Tris-HCl, 0.5mM EDTA, pH 9.0), and the A260/A280 optical density was measured. It was then stored at −80 °C until used.

### SNP selection and genotyping

We used the tag single nucleotide polymorphisms approach to determine the association between the SNPs and the high myopic subjects. To select the most representative SNPs to capture the majority of genetic variation, known SNPs encompassing the coding region of *IGF-1*, as well as the regions 9.8 kb upstream of the start codon and 8 kb downstream of the stop codon, were identified from Phase II+III in February, 2009 from the HapMap CHB (Han Chinese in Beijing, China) population. The tagger SNPs were selected by pairwise tagging using the tagger program in Haploview 4.2. Each tSNP had to meet the following criteria: r^2^>0.8 to capture 80% of genotype information in the region, and minor allele frequency (MAF)>10% in the Chinese Han population. Seven tSNPs were selected, and the average tag SNP was with r^2^=0.956.

All tSNPs were genotyped by the Beijing Genomics Institute (Shenzhen, China) using the MassArray platform and MALDI-TOF analysis (Sequenom, San Diego, CA).

### Statistical analysis

Hardy–Weinberg equilibrium (HWE) for genotypic distribution was determined by using the χ^2^ test for each group. Differences in the demographic features between case and control groups were tested for statistical significance by the χ^2^ test for dichotomous data and by the unpaired *t*-tests for continuous data. Genotypes were obtained by direct counting, and allele frequencies were calculated subsequently. Differences in the observed genotype and allelic frequencies were examined by the χ^2^ test for trend. Logistic regression analysis was performed by controlling age. The odds ratios (OR) and the corresponding 95% confidence intervals (CI) were calculated for associations concerning allele and genotype. Statistical analyses were performed on computer using the SPSS software (version 13.0; SPSS Science, Chicago, IL); and a p<0.05 was considered statistically significant. Bonferroni corrections for multiple comparisons were performed [16].

The haplotypes and LD blocks were inferred by the solid spine of LD with a minimum D′ of 0.8 in Haploview 4.2. The significance of the differences in the estimated haplotype frequencies between case and control groups were examined on Haploview 4.2 using χ^2^ tests, and haplotypes were corrected by using permutation test after running 50,000 times. Power calculations were performed using power and sample size calculations (PS; Version 3.0.43) [17].

## Results

### Clinical characteristics

Bilateral high myopia was present in 421 subjects (165 men, 256 women) with a mean age of 38.29±16.57 years (range: 5–80 years); the male to female ratio was 1.0:1.6. There were 401 control subjects (172 men, 229 women) with a mean age of 68.77±10.65 years (range: 40–95 years); the male to female ratio was 1.0:1.3. There was no significant difference for gender between the control and the patients. However, the mean ages were significantly different (p<0.001): the age in the control group was greater than that in the high myopia group. For subjects with high myopia, the spherical refractive errors of the right and left eyes were −14.57±5.60 D and −14.51±5.64D, respectively. The axial lengths of the right and left eyes were 28.33±2.26mm and 28.35±2.33mm, respectively (Table 1).

**Table 1:** Characteristics of the study population.

**Patients**	**High myopia cases**	**Extreme myopia cases**	**Control**	**p**
n	421	302	401	
Age, y(Mean±SD)	38.29±16.57	41.24±16.34	68.77±10.65	4.487E-16*
				1.887E-11**
**Sex, n(%)**				
Men	165(39.2)	116(38.4)	172(42.9)	0.281^#^
Women	256(60.8)	186(61.6)	229(57.1)	0.232^##^
**Axial length, mm**				
Right eye(Mean±SD)	28.33±2.26	28.98±2.27	23.13±0.67	
Left eye (Mean±SD)	28.35±2.33	29.02±2.35	23.07±0.68	
**Refraction, D**				
Right eye(Mean±SD)	−14.57±5.60	−16.54±5.26	0.39±0.82	
Left eye(Mean±SD)	−14.51±5.64	−16.39±5.47	0.42±0.80	

*p value between high myopia and control, **p value between extreme myopia and control were both tested by unpaired *t*-test. ^#^p value between high myopia and control, ^##^p value between extreme myopia and control were both performed based on the χ^2^ test.

### Power calculations

A power calculation indicated that a cohort of this size has 80% power to detect an association in the case group.

### Association study

The genotype counts and allele frequencies, associations, and odds ratios (ORs) for the 7 tSNPs in the high myopia and the control groups are shown in Table 2. The distributions of the genotypes for the 7 tSNPs were in HWE (p>0.05; Table 2). Among 7 *IGF-1* SNPs tested, three SNPs–rs5742629, rs12423791, and rs1457601–showed significant (p<0.05) differences in allele frequencies between the high myopia and control groups (p=0.021, p=0.024, p=0.040, respectively [Table 2]). After correction for age differences based on a logistic regression model, the differences were still significant (p=0.032, p=0.033, p=0.048, respectively). However, since multiple tests were undertaken in this analysis, we used the Bonferroni correction to identify tSNPs that showed significance at p<0.007. After this correction, no significant difference remained.

**Table 2:** The genotype distribution and allelic frequencies of polymorphisms in high myopia and control subjects.

**RefSNP ID**	**Genotype**	**Cases**	**Controls**	**OR (95% CI)**	**p-value**	**Allele**	**Cases**	**Controls**	**OR (95% CI)**	**p-value**
	n (%)	421	401			n (%)	842	802		
rs10860861	T/T	153 (36.3)	138 (34.4)	1	0.840	T	508 (60.3)	473 (59.0)	1	0.576
	T/C	202 (48.0)	197 (49.1)	0.902 (0.598–1.361)		C	334 (39.7)	329 (41.0)	0.945 (0.776–1.151)	
	C/C	66 (15.7)	66 (16.5)	0.975 (0.658–1.446)						
	HWE-p	0.96	0.76							
rs10860862	G/G	294 (69.8)	272 (67.8)	1	0.759	G	705 (83.7)	661 (82.4)	1	0.479
	G/T	117 (27.8)	117 (29.2)	0.771 (0.328–1.813)		T	137 (16.3)	141 (17.6)	0.911 (0.704–1.179)	
	T/T	10 (2.4)	12 (3.0)	0.833 (0.347–2.004)						
	HWE-p	0.68	0.89							
rs6214	G/G	99 (23.5)	100 (24.9)	1	0.686	G	403 (47.9)	400 (49.9)	1	0.414
	G/A	205 (48.7)	200 (49.9)	1.170 (0.796–1.719)		A	439 (52.1)	402 (50.1)	1.084 (0.893–1.315)	
	A/A	117 (27.8)	101 (25.2)	1.130 (0.813–1.572)						
	HWE -p	0.62	0.96							
rs5742629	A/A	128 (30.4)	157 (39.1)	1	0.031	A	478 (56.8)	500 (62.3)	1	0.021
	A/G	222 (52.7)	186 (46.4)	1.501 (0.988–2.281)		G	364 (43.2)	302 (37.7)	1.261 (1.035–1.536)	
	G/G	71 (16.9)	58 (14.5)	1.026 (0.689–1.527)						
	HWE -p	0.13	0.80							
rs12423791	G/G	219 (52.0)	241 (60.1)	1	0.064	G	608 (72.2)	618 (77.1)	1	0.024
	G/C	170 (40.4)	136 (33.9)	1.467 (0.838–2.569)		C	234 (21.8)	184 (22.9)	1.293 (1.034–1.616)	
	C/C	32 (7.6)	24 (6.0)	1.067 (0.600–1.896)						
	HWE -p	0.90	0.41							
rs35766	G/G	44 (10.5)	37 (9.2)	1	0.177	G	275 (32.7)	231 (28.8)	1	0.090
	G/A	187 (44.4)	157 (39.2)	0.771 (0.577–1.029)		A	567 (67.3)	571 (71.2)	0.834 (0.676–1.029)	
	A/A	190 (45.1)	207 (51.6)	0.772 (0.478–1.247)						
	HWE -p	0.84	0.36							
rs1457601	T/T	217 (51.5)	240 (59.9)	1	0.053	T	614 (72.9)	620 (77.3)	1	0.040
	T/A	180 (42.8)	140 (34.9)	0.889 (0.475–1.662)		A	228 (27.1)	182 (22.7)	1.265 (1.011–1.583)	
	A/A	24 (5.7)	21 (5.2)	1.264 (0.684–2.335)						
	HWE –p	0.09	0.92							

HWE-p indicates the p value of Hardy-Weinburg equilibrium. OR indicates odds ratio and CI indicates confidence interval which were performed by a logistic regression model. The p value was performed based on the χ^2^ test.

In relation of the two types of alleles that are present at each SNP (allele 1 and allele 2), statistical differences between groups were examined using their respective allele models of dominant and recessive (Table 3). For allele1 recessive model, rs5742629, rs12423791, and rs1457601 showed no significant difference between the high myopia and the controls (p=0.008, p=0.020, and p=0.017, respectively) after Bonferroni correction.

**Table 3:** Genotype frequencies (allele1 dominant and recessive model) for the 7 tSNPs in high myopia and control subjects.

	**Allele1 dominant model**					**Allele1 recessive model**				
	**Patients (n, %)**		**Control (n, %)**			**Patients (n, %)**		**Control (n, %)**		
**RefSNP ID**	**1/1+1/2**	**2/2**	**1/1+1/2**	**2/2**	p*	**1/1**	**1/2+2/2**	**1/1**	**1/2+2/2**	p*
rs10860861	355 (84.3)	66 (15.7)	335 (83.5)	66 (16.5)	0.760	153 (36.3)	268 (63.7)	138 (34.4)	263 (65.6)	0.563
rs10860862	411 (97.6)	10 (2.4)	389 (97.0)	12 (3.0)	0.584	294 (69.8)	127 (30.2)	272 (67.8)	129 (32.2)	0.535
rs6214	304 (72.2)	117 (27.8)	300 (74.8)	101 (25.2)	0.398	99 (23.5)	322 (76.5)	100 (24.9)	301 (75.1)	0.634
rs5742629	350 (83.1)	71 (16.9)	343 (85.5)	58 (14.5)	0.344	128 (30.4)	293 (69.6)	157 (39.2)	244 (60.8)	0.008
rs12423791	389 (92.4)	32 (7.6)	377 (94.0)	24 (6.0)	0.358	219 (52.0)	202 (48.0)	241 (60.1)	160 (39.9)	0.020
rs35766	231 (54.9)	190 (45.1)	194 (48.4)	207 (51.6)	0.063	44 (10.5)	377 (89.5)	37 (9.2)	364 (86.5)	0.556
rs1457601	397 (94.3)	24 (5.7)	380 (94.8)	21 (5.2)	0.770	217 (51.5)	204 (48.5)	240 (59.9)	161 (40.1)	0.017

*p value was performed based on the χ^2^ test.

**Figure 1:**
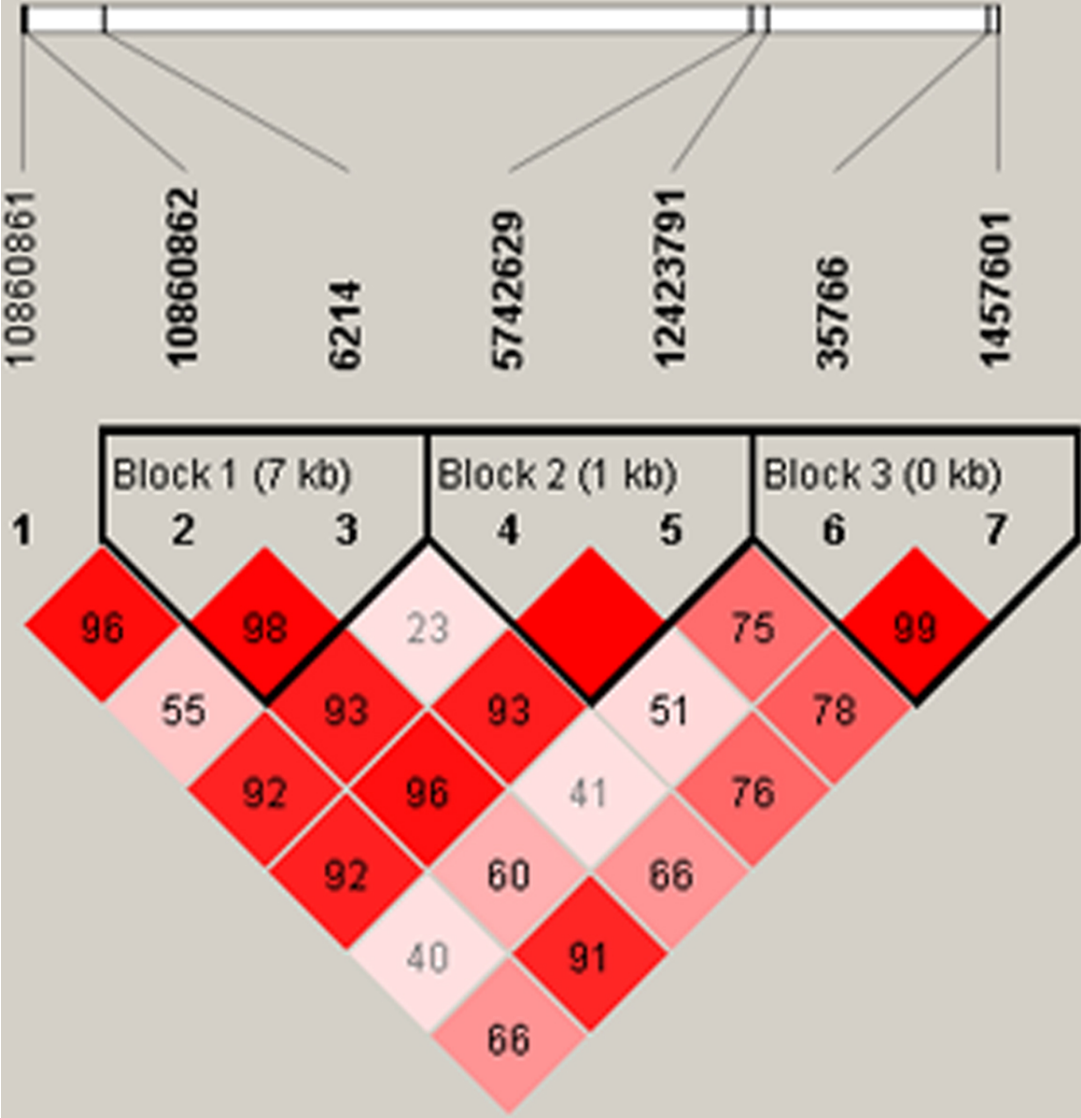
Three haplotype blocks of 7 tag SNPs of the *IGF-1* gene. Seven tag SNPs for *IGF-1* are shown in relation to the three haplotype blocks in HapMap CHB population, which were determined by the Haploview 4.2 program and the HapMap database. Darker shades represented stronger linkage disequilibrium.

The linkage disequilibrium block structure for *IGF-1* from the HapMap CHB population is indicated in Figure 1. The estimated haplotype frequencies in the high myopia group and the control group are shown in Table 4. In the subjects of our study, *IGF-1* rs10860862 and rs6214 at 12q23.2 were in strong LD (D'=0.98, r^2^=0.202), as well as rs5742629 and rs12423791 (D'=1.00, r^2^=0.495), rs35766 and rs1457601 (D'=0.99, r^2^=0.734; shown in Figure 1). We analyzed haplotype associations on the three blocks after removing haplotypes with frequencies less than 5%. There were no significant differences found between the high myopia and control groups after 50,000 permutations for multiple comparisons (Table 4).

**Table 4:** Haplotype analysis of the *IGF-1* gene in high myopia and control subjects.

**Block**	**Haplotype**	**Freq of case (%)**	**Freq of control (%)**	p value*	**OR (95%CI)**	P-permutation**
**Block 1**						
(rs10860862-rs6214)	GG	47.9	49.8	0.444	1	0.9620
	GA	35.9	32.8	0.184	1.138 (0.917–1.413)	0.6928
	TA	16.3	17.5	0.506	0.966 (0.735–1.270)	0.9826
**Block 2**						
(rs5742629-rs12423791)	AG	56.8	62.3	0.021	1	0.1346
	GC	27.8	22.9	0.024	1.330 (1.057–1.675)	0.1436
	GG	15.4	14.7	0.681	1.152 (0.872–1.523)	0.9986
**Block 3**						
(rs35766-rs1457601)	AT	67.3	71.1	0.010	1	0.4510
	GA	27.1	22.6	0.037	1.264 (1.007–1.586)	0.2109
	GT	5.6	6.2	0.566	0.943 (0.623–1.428)	0.9894

The asterisk indicates that differences in the estimated haplotype frequency were examined by the χ^2^ test. The double asterisk indicates that 50,000 permutations were performed. OR indicates odds ratio and CI indicates confidence interval which were performed by a logistic regression model.

However, after further stratification at −10.0D in both eyes, we found that rs12423791 showed significant differences between the extreme myopia and control groups in the allelic frequency (p=0.005; Table5) and the allele1 recessive model (p=0.004; Table 6). By controlling age under a logistic regression analysis, the difference was still showed (p=0.006). And after Bonferroni correction, the positive association was remained. Concerning haplotype associations on the three blocks, Block 2 (the haplotype GC of rs5742629-rs12423791) showed significant association with extreme myopia after 50,000 permutations (p=0.0334; Table 7).

**Table 5:** The genotype distribution and allelic frequencies of polymorphisms in extreme myopia and control subjects.

**RefSNP ID**	**Genotype**	**Cases**	**Controls**	**OR (95% CI)**	**p-value**	**Allele**	**Cases**	**Controls**	**OR (95% CI)**	**p-value**
rs5742629	n (%)	302	401			n (%)	604	802		
	A/A	87 (28.8)	157 (39.1)	1	0.016	A	341 (56.5)	500 (62.3)	1	0.026
	A/G	167 (55.3)	186 (46.4)	1.493 (0.939–2.374)		G	263 (43.5)	302 (37.7)	1.277 (1.030–1.583)	
	G/G	48 (15.9)	58 (14.5)	0.922 (0.596–1.425)						
	HWE -p	0.04	0.80							
rs12423791	G/G	149 (49.3)	241 (60.1)	1	0.016	G	425 (70.4)	618 (77.1)	1	0.005
	G/C	127 (42.1)	136 (33.9)	1.752 (0.970–3.165)		C	179 (29.6)	184 (22.9)	1.415 (1.113–1.798)	
	C/C	26 (8.6)	24(6.0)	1.160 (0.633–2.125)						
	HWE -p	0.89	0.41							
rs1457601	T/T	154 (51.0)	240 (59.9)	1	0.063	T	438 (72.5)	620 (77.3)	1	0.039
	T/A	130 (43.0)	140 (34.9)	1.336 (0.690–2.588)		A	166 (27.5)	182 (22.7)	1.291 (1.012–1.647)	
	A/A	18 (6.0)	21 (5.2)	0.923 (0.471–1.810)						
	HWE –p	0.16	0.92							

HWE-p indicates the p value of Hardy-Weinburg equilibrium. OR indicates odds ratio and CI indicates confidence interval which were performed by a logistic regression model. The p value was performed based on the χ^2^ test.

**Table 6:** Genotype frequencies (allele1 dominant and recessive model) for the 2 tSNPs in extreme myopia and control subjects.

	**Allele1 dominant model**					**Allele1 recessive model**				
	**Patients (n, %)**		**Control (n, %)**			**Patients (n, %)**		**Control (n, %)**		
**RefSNP ID**	**1/1+1/2**	**2/2**	**1/1+1/2**	**2/2**	p*	**1/1**	**1/2+2/2**	**1/1**	**1/2+2/2**	p*
rs12423791	276 (91.4)	26 (8.6)	377 (94.0)	24 (6.0)	0.180	149 (49.3)	153 (50.7)	241 (60.1)	160 (39.9)	0.004
rs1457601	284 (94.0)	18 (6.0)	380 (94.8)	21 (5.2)	0.678	154 (51.0)	148 (49.0)	240 (59.9)	161 (40.1)	0.019

* The p value was performed based on the χ^2^ test.

**Table 7:** Haplotype analysis of *IGF-1* in extreme myopia and control subjects.

**Block**	**Haplotype**	**Freq of case (%)**	**Freq of control (%)**	p value*	**OR (95%CI)**	P-permutation**
**Block 1**						
(rs10860862-rs6214)	GG	46.5	49.7	0.232	1	0.7773
	GA	37.0	32.7	0.098	1.209 (0.956–1.529)	0.4638
	TA	16.5	17.4	0.661	1.014 (0.752–1.367)	0.9977
**Block 2**						
(rs5742629-rs12423791)	AG	56.5	62.3	0.026	1	0.1541
	GC	29.6	22.9	0.005	1.426 (1.114–1.827)	0.0334
	GG	13.9	14.7	0.670	1.044 (0.764–1.426)	0.9981
**Block 3**						
(rs35766-rs1457601)	GA	66.1	71.1	0.042	1	0.2409
	AT	27.5	22.6	0.037	1.308 (1.002–1.673)	0.2138
	AA	6.5	6.2	0.875	1.112 (0.718–1.723)	1.0000

The asterisk indicates that differences in the estimated haplotype frequency were examined by the χ^2^ test. The double asterisk indicates that 50,000 permutations were performed. OR indicates odds ratio and CI indicates confidence interval which were performed by a logistic regression model.

## Discussion

A more comprehensive way to examine the *IGF-1* gene is through a tSNP approach that efficiently encompasses all the known common variants and most of the unknown common variants in the gene. Our tSNP approach identified 7 tSNPs (rs10860861, rs10860862, rs6214, rs12423791, rs35766, rs1457601, and rs5742629) that efficiently tagged common variants with a MAF>0.1 in the *IGF-1* gene (based on HapMap CHB population data), including rs6214, which was the first to be reported as having significant association with high myopia in a Caucasian family-based data set [14]. In this study, we identified rs12423791, which is located in intron region of *IGF-1*, as associated with extreme myopia in our population, while there were no significant differences in the genotypic distributions and allele frequencies of the 7 tSNPs between the high myopia and controls after Bonferroni correction. Furthermore, since association studies that incorporate linkage disequilibrium information may offer more powerful analysis than individual SNP analysis to identify causal genetic variants underlying complex disease [18], we analyzed haplotype associations on the 7 tSNPs in relation to the three haplotype blocks based on the HapMap CHB population by the Haploview 4.2 program. Our data demonstrated an association of Block 2 (rs5742629-rs12423791) with extreme myopia, whereas there was no statistically significance between the high myopia and control group after Bonferroni correction. Hence, selecting a more severe degree of myopia with the purpose of providing a higher likelihood of strong genetic background as entry criterion for cases plays an important role in the field of myopia genetic association studies.

In addition, the mean ages of the controls were significantly different between high myopia and control groups by unpaired *t*-tests, and the mean age in the control group was obviously greater than that in the high myopia cases. However, this should not impact our conclusion since high myopia is an early onset disease, usually occurring no later than the puberty years. Also, the degree of myopia normally increases with age. Although genetic factors are important in the development of myopia, especially high myopia [13], the large increase in incidence over the past 50 years suggests a much stronger impact of environmental factors in the younger generation [19]. When we enrolled controls of ≥40 years of age rather than general population, it implied that the subjects were unlikely to be high myopia patients due to environmental factors. Therefore, the more stringent criteria for the control subjects in our study enabled us to get a higher relative risk value in the current sample size, and allowed minimization of potential effects from general-population-based controls.

*IGF-1* has been considered as a target candidate gene of genetic association studies for numerous human diseases, including diabetes, diabetic retinopathy (DR), cancer susceptibility, osteoarthritis, bodyweight, growth, menarche, and longevity [20]. It has been implicated in ocular diseases such as proliferative DR, retinopathy of prematurity (ROP), and age-related macular degeneration (AMD) [21-25]. In a recent study, *IGF-1* polymorphisms were tested for possible association with myopia, and the SNP rs6214 showed significant association with the high-grade and any-myopia phenotypes [14].

These findings were in line with recent evidence by an experimental myopia model that showed that *IGF-1* promotes ocular growth and axial myopia. In the present study, we used an effective SNP selection strategy, definitive ascertainment criteria, to validate and replicate this finding in the Chinese population. However, in the high myopia group we failed to replicate this result. Thus far, the results are very limited for replication studies that have attempted to verify myopia susceptibility genes. This is not a surprise since it is common in complex diseases that the significant association signals in one investigation appear to be negative in other analyses. There may be several reasons for such phenomena, including different genetic backgrounds between populations and minor diversities in sample collection, clinical measurement, and statistical analysis [15]. In this study, the minor allele frequencies of the SNP rs12423791 among Europeans, Han Chinese, and Japanese are 0.009, 0.25, and 0.209, respectively, based on HapMap data, while the SNP rs6214 minor allele frequencies are 0.421, 0.467, and 0.568, respectively. Therefore, the different ethnicities between the two studies may have been the main contribution to the difference in the result. Besides, while the research of Metlapally et al. [14] found three SNPs (rs10860860, rs2946834, and rs6214) out of 13 *IGF-1* tSNPs showed p<0.05, after Bonferroni correction for multiple testing only rs6214 showed significant association with myopia. In our study, we also detected three SNPs–rs5742629, rs12423791, and rs1457601–present at p<0.05 significant in the genotypic distributions and allele frequencies between high myopia and control subjects. However, significant associations for this study would require an adjusted p-value (<0.007) through Bonferroni correction. Therefore, no association was found at this level for multiple comparisons in the high myopia group. The Bonferroni correction is commonly applied in association studies to evaluate the significance of multiple testing of SNPs, but it is usually too conservative [16]. We realize that this correction is a limitation and it may lead to loss of significant findings. For this reason, a much larger case–control study of the significance of tSNPs and appropriate multiple-correction criteria were applied, which would be more definitive way to determine a real association in a separate population.

High myopia is considered as a complex and multigenic disease involving several overlapping signaling pathways, each one mediated by a group of distinct genetic profiles. Therefore, studying genetic polymorphisms that are related to the mechanisms of myopia can help to further clarify the relationship between genetics and myopia. In this study, we found that *IGF-1* polymorphism may be associated with extreme myopia in the Chinese population and should be investigated further.
